# A SCOPING REVIEW: INNOVATIVE APPROACHES TO ACHIEVE HEALTH EQUITY FOR OLDER ADULTS

**DOI:** 10.1093/geroni/igae098.0685

**Published:** 2024-12-31

**Authors:** Meshack Otewa, Priscilla Carmiol-Rodriguez

**Affiliations:** University of Washington, Seattle, Washington, United States; University of Washington, Washington, United States

## Abstract

The purpose of this scoping review was to comprehensively examine innovative approaches proposed or implemented to achieve health equity among older adults. Health equity, defined as ensuring optimal health for all, regardless of various determinants, remains elusive among older adults, a population experiencing significant growth and associated health challenges. Following PRISMA-ScR guidelines, the review searched PubMed, CINAHL, and Embase for original research articles from 2013 to 2023, yielding 47 eligible articles. Findings reveal a range of innovative approaches, defined as new care models improving outcomes and value for health equity. Health disparities are escalating, particularly in chronic conditions like cancer, mental health, HIV-AIDS, pain, and nutritional issues among older adults. Approaches identified include leveraging digital health technology, ensuring access to health insurance (e.g., US Medicaid) with a focus on non-discriminatory access for individuals who identify as LGBTQI, fostering age-friendly health systems, conducting health economics research, expanding public health initiatives for older adults, integrating cultural considerations into health practices, and employing a multimodal approach to translating research into policy and practice. The need for standardized categories for these approaches emphasizes the need for systematic effectiveness assessment. The results of this scoping review suggest that while innovative approaches may be novel, incorporating new values into existing systems is crucial. As health disparities persist and expand, the timely innovation of techniques becomes critical to narrowing the gap and achieving health equity among older adults. The identified methods are a valuable foundation for future program development and system reformulation.

